# Nourish the mind: protocol for an umbrella review on nutrients and bioactive food components supporting brain health across the lifespan

**DOI:** 10.1186/s13643-026-03250-6

**Published:** 2026-06-22

**Authors:** Berivan Ece, Corinne H. Miller, Xiaoran Liu, Patricia Bucko, Colby J. Vorland, Chwan-Li Shen, Lauren Roberson

**Affiliations:** 1 Feinberg School of Medicine, Department of Medical Social Sciences, Northwestern University, Chicago, IL USA; 2 Feinberg School of Medicine, Galter Health Sciences Library & Learning Center, Northwestern University, Chicago, IL USA; 3 Rush University Medical Center, Rush Institute for Healthy Aging, Chicago, IL USA; 4 Department of Epidemiology and Biostatistics, Indiana University School of Public Health-Bloomington, Bloomington, IN USA; 5 Department of Pathology, Texas Tech University Health Sciences Center, Lubbock, TX USA; 6 College of Education and Human Ecology, Department of Human Sciences, The Ohio State University, Columbus, OH USA

**Keywords:** Nutrition, Nutrients, Bioactive food components (BFCs), Brain health, Cognition, Cognitive health, Umbrella review, Evidence synthesis

## Abstract

**Background:**

Brain health is the state of optimal brain functioning across cognitive, sensory, social-emotional, behavioral, and motor domains, supporting individuals to achieve their full potential at every stage of life. Diet is a modifiable factor that plays a fundamental role in supporting brain health. Although many systematic reviews have examined individual nutrients or bioactive food components (BFCs) in relation to cognitive and related brain health outcomes, no comprehensive umbrella review has yet synthesized this evidence across nutrients, BFCs, life stages, intake levels, and brain health outcomes. To our knowledge, this will be the first umbrella review to systematically map and synthesize evidence across nutrients, BFCs, life stages, intake levels, and brain health domains in generally healthy individuals free from major chronic medical, neurological, psychiatric, and neurodegenerative conditions.

**Methods:**

This umbrella review will follow the Joanna Briggs Institute (JBI) methodology. A comprehensive search will be conducted in Embase (Elsevier), MEDLINE (Ovid), Web of Science (Clarivate), PsycINFO (EBSCO), CINAHL (EBSCO), and Cochrane Database of Systematic Reviews (Wiley) to identify systematic reviews published between January 1, 2016 and March 2, 2026 that meet eligibility criteria. Two trained reviewers will independently screen titles/abstracts and full texts, with data extraction performed only from the retained reviews. The methodological quality of included reviews will be appraised using the AMSTAR-2 (A Measurement Tool to Assess Systematic Reviews 2), and the overlap of primary studies across reviews will be evaluated using the Corrected Covered Area (CCA) method. The results will be reported in adherence to the Preferred Reporting Items for Overviews of Reviews (PRIOR) guidelines.

**Discussion:**

This umbrella review will provide an integrated summary of existing systematic review evidence on nutrients and BFCs in relation to cognitive and related brain health outcomes in generally healthy populations. Findings will guide the development of age-specific, evidence-based dietary strategies to promote optimal brain health across the lifespan and support public health recommendations and future dietary guidelines.

**Systematic review registration:**

PROSPERO CRD420251186632.

**Supplementary Information:**

The online version contains supplementary material available at https://doi.org/10.1186/s13643-026-03250-6.

## Strengths and limitations of this study

- To our knowledge, this will be the first umbrella review to comprehensively synthesize evidence on nutrients and bioactive food components (BFCs) linked to cognitive and related brain health outcomes across the entire lifespan in generally healthy individuals.
- This review will follow the Joanna Briggs Institute (JBI) methodology for umbrella reviews and adhere to the Preferred Reporting Items for Overviews of Reviews (PRIOR) guidelines to ensure methodological rigor and transparency.
- Where possible, intake levels of nutrients and BFCs will be extracted and reported by age group to identify potential developmental or age-related differences.
- A key strength is the distinct classification of evidence, differentiating between outcomes explicitly validated for brain health versus those extrapolated from general physiological health markers.
- We will also examine the degree of redundancy among included systematic reviews using the corrected covered area (CCA), allowing us to contextualize and report the evidence overlap.
- Findings will provide evidence to inform dietary guidelines and policy development aimed at promoting optimal brain health.
- The identification and synthesis of data will be limited to systematic reviews, excluding peer-reviewed publications other than systematic reviews and other sources such as grey literature (e.g., dissertations, non-formally published studies), and study protocols.
- Restricting inclusion to systematic reviews published in English since 2016 and variability in how “brain health” is defined may limit comparability and generalizability of findings.

## Background

The World Health Organization (WHO) defines *brain health* as “the state of brain functioning across cognitive, sensory, social-emotional, behavioral, and motor domains, allowing a person to realize their full potential over the life course, irrespective of the presence or absence of disorders” [1]. Diet is a modifiable factor that plays a fundamental role in improving brain health. Large-scale studies, such as the COSMOS (COcoa Supplement and Multivitamin Outcomes Study) [2] and the MIND (Mediterranean-DASH Intervention for Neurodegenerative Delay) [3] trials, have assessed the effects of multivitamins and dietary patterns on brain health. Although some findings remain mixed, results from the recent randomized controlled trial, the U.S. POINTER (Protect Brain Health Through Lifestyle Intervention to Reduce Risk) [4] study, showed that lifestyle factors such as exercise and adherence to the MIND diet significantly improved global cognition in older adults. Whether evidence exists to set policy for individual nutrients and brain health outcomes remains uncertain.

While dietary patterns have been extensively investigated, it is also important to consider how current nutrient reference values are determined. Existing nutrient reference values such as *Dietary Reference Intakes (DRIs)* primarily focus on *preventing* nutrient deficiencies rather than *promoting* cognitive and related brain health outcomes. For example, the *adequate intake (AI)* level for *alpha-linolenic acid (ALA)* is established independently of its impact on brain health outcomes, despite its recognized importance for brain health [5]. Similarly, nutrients such as omega-3 and omega-6 fatty acids, B vitamins, fat-soluble vitamins (e.g., vitamins D and E), and minerals including magnesium and zinc have been reported to promote brain health [6, 7], yet their DRIs do not currently incorporate evidence from brain health outcomes [5].

To promote brain health, it is essential to define optimal nutrition through the lens of cognitive and related brain health outcomes, rather than physiological sufficiency alone [8]. Although there are few, if any, reviews to our knowledge that specifically focus on nutrition in healthy populations and brain health outcomes, existing literature has examined the relationship between nutrition and neurodegeneration [9, 10], as well as mental health outcomes [11, 12]. While numerous systematic reviews have examined the relationship between nutrition and brain health [13, 14], the existing literature is scattered and still lacks a comprehensive evidence synthesis across nutrients, BFCs, brain health outcomes, and age groups. To address this need, we propose an umbrella review of the systematic reviews (with or without meta-analysis) published in the last decade, focusing on specific nutrients and BFCs, including their optimal intake levels in healthy individuals (i.e., those without major chronic medical, neurological, psychiatric, or neurodegenerative conditions) across the lifespan. This comprehensive synthesis aims to identify nutrients and BFCs most strongly associated with brain health outcomes and inform the development of evidence-based recommendations, such as the current and future iterations of the *Dietary Guidelines for Americans* [15]. Where available, we will report intake levels and indicate whether these levels are recommended for general health or explicitly for brain health.

Finally, most studies examining the relationship between nutrition and cognition focus on adults aged 65 and older [13, 16]. However, emerging evidence suggests that midlife, and even earlier stages, may serve as sensitive developmental windows, offering promise for preventive strategies [17]. To capture these potential windows and age-specific brain health outcomes, data extracted in this umbrella review will be stratified by age groups: toddlers (13–23 months), children (2–8 years), adolescents (9–18 years), adults (19–59 years), and older adults (60 years and above). These age categories were chosen to balance alignment with the *Dietary Guidelines for Americans (2020–2025) *[15] life stages and the need for finer granularity in developmental windows relevant to brain health. While the Dietary Guidelines group children and adolescents together and define older adults as 60 years and above, the selected age groups allow for more precise examination of nutrient-brain relationships across sensitive developmental periods [15]. Focusing on age-specific brain health outcomes within a lifespan framework is a key strength of this review. Because brain health outcomes and underlying neurobiological mechanisms differ across developmental stages [18, 19], this approach will help clarify how nutrient requirements—and their effects on cognitive function—may vary from toddlers through older adulthood.

## Objective and research questions

The objective is to synthesize existing evidence from systematic reviews (with or without meta-analysis) on nutrients and BFCs in relation to brain health outcomes across the lifespan, excluding infancy (0–12 months) and pregnancy. By mapping and summarizing findings across life stages, nutrient categories, and brain health outcomes, we will identify which nutrients and BFCs show the strongest and most consistent relationships with brain health in healthy individuals. In addition, we will summarize reported intake levels and indicate whether these levels were recommended for general health or explicitly linked to brain health outcomes. Together, these findings will help clarify current evidence gaps and research directions to promote brain health in healthy individuals across the life course.

We developed our research questions using the *Population–Exposure–Outcome (PEO) *[20] framework as recommended for umbrella reviews. The research questions are presented below, and the corresponding PEO elements are provided in Table 1.

1. How is “brain health” defined, operationalized, and measured across existing systematic reviews?
2. Which nutrients and BFCs have been identified in systematic reviews as being associated with specific cognitive and related brain health outcomes (e.g., memory, attention, executive function, sensory processing, motor function) in healthy individuals across the lifespan (excluding pregnancy and infancy)?
3. For each identified nutrient and BFC, what intake levels are reported in systematic reviews, and how do these compare with current *DRIs, AI levels, Recommended Dietary Allowances (RDAs), and Estimated Average Requirements (EARs)*?
4. What key evidence gaps have been identified in existing systematic reviews that should guide future research and policy on diet and brain health?

**Table 1:** PEO framework for the umbrella review

PEO element	Explanation
Population (P)	Generally healthy individuals (excluding infants and pregnant women; free from major chronic medical, neurological, psychiatric, and neurodegenerative conditions)
Exposure (E)	Nutrients and BFCs (e.g., vitamins, minerals, omega-3 fatty acids, polyphenols, flavonoids, other dietary compounds)
Outcome (O)	Cognitive and related brain health outcomes (e.g., memory, attention, executive function, sensory processing, motor function, and overall brain health measures used in included systematic reviews)

## Methods/design

An umbrella review methodology will be used to identify and evaluate published systematic reviews on the nutrients and BFCs supporting brain health in healthy individuals across the lifespan. Umbrella reviews summarize high-level evidence from multiple systematic reviews and extract relevant findings from the highest-quality primary studies [21–23]. This provides a broad overview of the literature and highlights gaps in the evidence.

This review will employ the *Joanna Briggs Institute (JBI)* methodology [24] and results will be reported in adherence to the *Preferred Reporting Items for Overviews of Reviews (PRIOR) *[25] guidelines to ensure transparency, reproducibility, and methodological rigor (see Supplementary material 1 for the completed PRIOR checklist). In addition, this protocol has been registered in PROSPERO (CRD420251186632) for further methodological transparency.

### Eligibility criteria

Systematic reviews (with or without meta-analysis) will be included in this umbrella review according to predefined eligibility criteria, covering population, exposure, outcomes, study design, language, and publication date.

#### Population

Although brain health is a broad construct that includes cognitive function across the lifespan and may be affected in neurodegenerative and mental health conditions, this umbrella review focuses specifically on brain health in generally healthy populations. We intentionally exclude systematic reviews targeting neurodegenerative diseases (e.g., Alzheimer’s disease), mental health disorders (e.g., depression), and clinical cognitive impairment (e.g., Mild Cognitive Impairment [MCI]), because these conditions reflect disease-specific pathophysiology that may substantially alter nutrient–brain relationships. Including such populations would introduce substantial clinical heterogeneity and limit interpretability for primary prevention and population-level dietary guidance. Therefore, we will include systematic reviews (with or without meta-analysis) that focus on *generally healthy individuals,* which are operationally defined as persons who (1) do not have a diagnosed neurological, psychiatric, neurodegenerative, or other major chronic medical disorder (e.g., cardiovascular disease, diabetes, cancer) and (2) do not present with clinical or preclinical cognitive impairment, including MCI or Subjective Cognitive Decline (SCD). Included reviews must have targeted *generally healthy populations as defined by their inclusion criteria* and where unclear, we will classify them as mixed populations and record this.

For the purposes of this umbrella review, infancy and pregnancy were excluded because these life stages are characterized by distinct and rapidly changing physiological, metabolic, and neurodevelopmental demands, including substantially different nutrient requirements, growth trajectories, and hormonal environments [26]. These factors fundamentally alter nutrient–brain relationships and would introduce additional heterogeneity that is not comparable to other life stages included in this review. In addition, evidence in these populations is typically evaluated within specialized nutritional frameworks (e.g., infant feeding guidelines and pregnancy-specific dietary reference standards), which differ substantially from general adult dietary guidance and would limit comparability for population-level recommendations [27, 28]. Accordingly, systematic reviews focusing exclusively on infants (0–12 months), pregnant women, clinical populations with neurodegenerative diseases (e.g., Alzheimer’s disease), mental health disorders (e.g., depression), preclinical cognitive impairment (e.g., MCI, SCD), or other chronic disease populations will be excluded. Reviews based on animal or in vitro studies will also be excluded, as the aim is to synthesize evidence relevant to human populations and typical brain health rather than disease-specific or mechanistic outcomes.

#### Exposure

Only systematic reviews that examine nutrients or BFCs supporting brain health will be included. For the purpose of this review, BFCs are defined and categorized following the taxonomy described by Kussmann et al. [29]. Reviews focusing on caffeine as the only nutrient will be excluded. Caffeine is excluded as a standalone exposure due to its unique pharmacological profile as a psychoactive stimulant and its frequent co-consumption with other bioactive compounds (e.g., polyphenols in coffee and tea), which introduces substantial confounding in isolating nutrient-specific effects [30]. In addition, most systematic reviews assessing caffeine evaluate mixed beverage or dietary sources rather than caffeine in isolation, limiting comparability with other nutrients and bioactive food components defined in this review. The aim of this umbrella review is to synthesize evidence on nutrients and BFCs with relatively distinct nutritional roles in supporting brain structure and function; therefore, including caffeine would introduce heterogeneity in exposure classification and outcome interpretation. Future work may consider dedicated synthesis of caffeine and other commonly consumed neuroactive dietary compounds given their widespread use and potential cognitive effects [31].

#### Outcomes

The primary outcomes for this umbrella review are cognitive and related brain health outcomes in generally healthy populations, including domains such as memory, attention, executive function, sensory processing, and motor function. Systematic reviews that focus exclusively on cognitive and other brain health outcomes associated with neurodegenerative diseases, mental health disorders, pregnancy, or other clinical disease-specific conditions will be excluded.

#### Study design

Only systematic reviews (with or without meta-analysis) published in peer-reviewed journals will be included in this review in addition to preprints of systematic reviews. Preprints will be included to ensure comprehensive coverage of recent evidence, particularly in this rapidly evolving field. To mitigate risk, all preprints will undergo the same AMSTAR-2 (A Measurement Tool to Assess Systematic Reviews 2) [32] appraisal as peer-reviewed reviews, and their non-peer-reviewed status will be explicitly noted in the synthesis. All other sources such as research articles, grey literature, books or book chapters, and study protocols will be excluded.

#### Language

Only English-language systematic reviews will be included, as this was specified in the funding call. We recognize that this may introduce some language bias, but it allows us to follow the project requirements and stay within the resources available to the team.

#### Publication date

This umbrella review will include systematic reviews published between January 1, 2016 and March 2, 2026. Restricting inclusion to recent systematic reviews is intended to ensure that the synthesized evidence reflects contemporary randomized controlled trial data, updated methodological standards, and current best practices in evidence synthesis, rather than potentially outdated conclusions. This is particularly important given the rapid evolution of the nutrition and brain health field over the past decade, including substantial growth in randomized controlled trials and advances in methodological approaches.

The eligibility criteria for systematic reviews to be included in this umbrella review are summarized in Table 2. Only reviews that meet these criteria will be considered.

**Table 2:** Inclusion and exclusion criteria for the umbrella review

Criteria	Inclusion	Exclusion
Population	Healthy individuals aged ≥ 13 months	Infants (< 13 months) Pregnant women Neurodegenerative diseases (e.g., Alzheimer’s) Mental health disorders (e.g., depression) Major chronic medical condition Animal or in vitro studies Clinical populations
Exposure	Nutrients or BFCs related to brain health (e.g., vitamins, minerals, omega-3 fatty acids, polyphenols, flavonoids)	Reviews focusing only on caffeine as a nutrient
Outcomes	Cognitive and related brain health measures in healthy populations (e.g., memory, attention, processing speed)	Outcomes specific to neurodegenerative or mental health disorders Clinical disease-specific outcomes
Study design	Systematic reviews (with or without meta-analysis) Preprints of systematic reviews	All other sources (e.g., research articles, grey literature, books/chapters, study protocols)
Language	Published only in English	Non-English publications

### Search strategy

To identify systematic reviews (with or without meta-analysis) that examine nutrients and BFCs promoting brain health, we will conduct a comprehensive search of peer-reviewed literature published over the past 10 years. Searches will be performed in Embase (Elsevier), MEDLINE (Ovid), Web of Science (Clarivate), PsycINFO (EBSCO), CINAHL (EBSCO), and the Cochrane Database of Systematic Reviews (Wiley). The search strategies will be developed by our co-investigator, who is a librarian from Northwestern University’s Galter Health Sciences Library & Learning Center, in consultation with subject matter experts on our team, to ensure comprehensive and accurate identification of relevant studies. Subject headings and keywords will be utilized to locate literature on BFCs and brain health. The categorization of BFCs was based on the classification framework provided by Kussmann et al. (2023) [29] ensuring a comprehensive inclusion of various chemical classes such as polyphenols, alkaloids, and bioactive lipids. A filter will be applied to omit literature focusing solely on animal populations. Lastly, validated filters will be adapted to identify systematic reviews [33, 34]. Our full search strategy for Ovid MEDLINE is provided in Supplementary Table S1.

#### Dry-run screening

To transition from training to actual screening, the research team completed an initial dry-run screening using a preliminary set of 30 records. These records were identified based on the search strategy developed by a university librarian (CHM), who serves as a co-investigator and the second author of this study. All research assistants underwent comprehensive training led by the first author and completed this practice phase to apply the eligibility criteria. They independently evaluated titles and abstracts, followed by feedback on their decisions and justifications. During dry-run screening, the correct decision rates for research assistants ranged from 86.7 to 93.3%, demonstrating strong alignment with the study’s eligibility criteria. This process ensured that the team has achieved the necessary proficiency and consensus to begin the formal screening phase with high procedural consistency. While the primary goal was reviewer readiness, this dry-run also allowed minor secondary refinements to the eligibility criteria.

#### Title and abstract screening

Search results will be deduplicated, then uploaded to Covidence [35] for screening. Each systematic review will be screened by two trained reviewers independently. Any record deemed potentially relevant by either reviewer will be flagged for conflict resolution. Conflicts will be resolved by a third reviewer, and based on this decision, records will be either included or excluded from the full-text review.

#### Full text review

At this stage, two independent reviewers will screen the full-text records to determine whether each systematic review should be included or excluded based on the eligibility criteria. Any disagreements will be resolved by a third reviewer. To assess inter-rater reliability, Cohen’s κ statistic [36] will be calculated for both the title/abstract and full-text screening stages.

#### Full text extraction

Two independent reviewers will extract data from each systematic review, and inter-rater reliability will be assessed and evaluated. Disagreements between reviewers will be resolved through joint discussion or with an independent third reviewer.

### Data extraction

The data extraction form will include the information listed below:

A. General systematic review characteristics
  1. Publication details (e.g., title, doi number, publication year and country)
  2. Funding source and potential conflicts of interest
B. Methodology
  3. Aim of the systematic review
  4. Reporting guidelines applied, if any
  5. Design of studies included in the review (e.g. randomized controlled trials, controlled prospective cohort, repeat cross sections)
  6. List and number of databases searched
  7. Search period
  8. Number of initial records (before screening and exclusions)
  9. Number of eligible studies
  10. Total number of participants across all included studies
  11. Method of synthesis (meta-analysis or narrative)
  12. Tool(s) used by the review authors to assess included primary studies (e.g., Cochrane Risk of Bias Tool, Newcastle–Ottawa Scale)
  13. Inclusion and exclusion criteria
C. Population and context details:
  14. Geographic location of included studies
  15. Age range included in the systematic review
  16. Life stages represented
    - Life stages according to *Dietary Guidelines for Americans (2020–2025) *[15]
    - Life stages according to brain development [37, 38]
  17. Health status of the included populations (e.g., healthy, at-risk)
D. Exposure details:
  18. Specific nutrients or BFCs focused in the review
  19. Comparison group (e.g., placebo, patients, usual care), if applicable
  20. Method of dietary intake assessment (e.g., self-report such as 24-h recall, food diary, food frequency questionnaire; or biometric measures such as blood, urine, skin carotenoids)
  21. Intake levels reported
  22. Frequency of intake (e.g., once daily, twice a week)
  23. Duration of exposure, follow-up period if applicable
  24. Dosage for supplements, if applicable
  25. Target age group of the intake level (i.e., children, adolescent, adults, older adults)
  26. Intervention type, if applicable (e.g., supplement vs. whole food, controlled feeding)
E. Outcomes details:
  27. Definition of brain health
  28. Specific brain health outcomes targeted (e.g., memory, attention, processing speed)
  29. Brain health measures
  30. Type of brain health measure (e.g., subjective test vs neuroimaging outcome vs biomarker)
F. Overall findings:
  31. Key findings (including direction and strength of associations)
  32. Implications for future research
  33. Main conclusions
  34. Limitations (as reported in the review).

### Quality assessment

Two reviewers will independently assess the methodological quality of all included systematic reviews using the *AMSTAR-2 *[32]. This validated critical appraisal tool is designed to evaluate systematic reviews that include randomized and/or non-randomized studies of healthcare or nutrition-related interventions. The AMSTAR-2 checklist consists of 16 items that assess critical domains such as study selection, data extraction, risk of bias, and synthesis methods (see Supplementary material 2). Each item will be rated as “yes” (criterion fully met), “partial yes” (criterion partially met), or “no” (criterion not met).

Based on AMSTAR-2 guidance, the overall methodological quality of each review will be categorized as high, moderate, low, or critically low. In addition, where available, authors’ assessments of the certainty or quality of evidence (e.g., those based on *Grading of Recommendations, Assessment, Development and Evaluation* (GRADE) [39] approach will be extracted to provide further insight into the overall strength and consistency of evidence across reviews.

Prior to the formal appraisal, all reviewers will complete a practice session using a subset of the included systematic reviews to ensure consistent application of the AMSTAR-2 criteria. Any discrepancies between reviewers will be resolved through either team discussion or a third reviewer.

For transparency, the results of the AMSTAR-2 assessment will be summarized in a table including item-level ratings and overall confidence classifications. Aggregate findings will be described narratively to highlight common methodological strengths, weaknesses, and patterns across reviews.

#### Assessment of overlap

To assess the extent of overlap among the systematic reviews included in our umbrella review, we will compute the *Corrected Covered Area (CCA)* as described by Pieper and colleagues (2014) [40]. The CCA measures the number of primary studies common across different systematic reviews, providing an estimate of redundancy. It accounts for both the number of included reviews and the total number of primary studies, resulting in a value ranging from 0 (no overlap) to 1 (complete overlap). Based on established interpretation thresholds, a CCA of less than 5% signifies slight overlap; 6–10% indicates moderate overlap, 11–15% reflects high overlap, and more than 15% denotes very high overlap. The findings will be summarized in a matrix that illustrates the distribution of primary studies across the reviews and will be presented both numerically and visually. This analysis will help contextualize the degree of evidence redundancy and inform interpretation of the synthesized results.

### Data analysis and presentation of results

The results of this umbrella review will be reported in accordance with the PRIOR [41] guidelines. A comprehensive descriptive synthesis will be conducted to summarize the findings from the final set of systematic reviews including population demographics, life stages, and brain health domains across reviews. Extracted data will be tabulated and narratively summarized to capture key characteristics, methodological quality, and outcomes.

#### Brain health definition and outcomes

For this umbrella review, brain health will be operationalized according to the WHO definition [1], which encompasses functioning across *cognitive*, *sensory*, *social-emotional*, *behavioral*, and *motor domains*, allowing individuals to realize their full potential across the life course, irrespective of the presence or absence of disorders. Systematic reviews included in this umbrella review will be categorized based on how their definitions of brain health align with the domains included in this framework. Although we draw on the WHO definition of brain health, we recognize the heterogeneity in how brain health outcomes are defined and measured across systematic reviews. Given this variability across reviews in how brain health outcomes are operationalized and measured, we will not conduct formal quantitative pooling or meta-analysis. Instead, outcomes will be mapped and narratively synthesized. To ensure consistency in screening and data extraction, we will apply an operational outcome framework in which brain health outcomes will be grouped into the following predefined domains:

1. Cognitive function (e.g., memory, attention, executive function, processing speed, learning, global cognition)
2. Sensory function (e.g., vision, hearing, olfaction) when explicitly linked to brain or neural processing
3. Motor function (e.g., balance, coordination, gait) when interpreted as indicators of neurological function
4. Social–emotional or behavioral regulation (e.g., emotion recognition, social cognition, self-regulation), provided they are assessed as outcomes of brain functioning rather than mental health disorders.

If, during data extraction, we identify additional brain health–related outcome domains that are not captured within these predefined categories yet appear frequently across included systematic reviews, we will establish new emergent categories. Any such categories will be clearly described and justified in the final synthesis.

#### Nutrients, BFCs, and intake levels

Included systematic reviews will be grouped by nutrients or BFCs and, where applicable, by dietary patterns (e.g., Mediterranean, DASH, MIND). Because nutrient intake levels are reported inconsistently across systematic reviews (e.g., mg/day, g/day, servings, % energy, biomarker concentrations), we will harmonize intake data where feasible. When necessary, units will be converted using standard nutritional reference values or reported as “non-standardized” when conversion is not possible. Reviews lacking quantifiable intake information will still be included but categorized separately. This approach enhances comparability while maintaining transparency.

#### Life stages

Systematic reviews will also be organized by life stages: toddlers (13–23 months), children (2–8 years), adolescents (9–18 years), adults (19–59 years), and older adults (60 years and above) [15]. Defining these age groups is critical, as nutrient requirements, brain development trajectories, and responsiveness to interventions differ across life stages [42], enabling the review to provide age-specific evidence that may inform the development of tailored dietary guidelines and recommendations. Although recent evidence suggests important neurobiological transitions across adulthood, including around the early fourth decade of life [38], broader adult age categories were retained to support consistency with dietary guideline frameworks and comparability across included systematic reviews with heterogeneous age groupings. Where sufficient data are available, age-related patterns across adulthood will also be explored narratively.

Where studies include broader age ranges, data will be assigned to the most appropriate life stage based on the reported mean or median age. If age-specific data are not separable, the study will be classified according to the predominant age group. This categorization enables age-stratified synthesis while aligning with developmental stages and dietary guideline structures.

### Patient and public involvement

Patients and the public will not be involved in the design, conduct, reporting, or dissemination plans of this study.

## Discussion

This protocol manuscript outlines the methodological framework for an umbrella review. By integrating findings from existing systematic reviews (with or without meta-analysis), we will provide a high-level synthesis of nutrients and BFCs supporting cognitive and broader brain health outcomes in healthy individuals, including reported intake levels across different life stages. Findings from this umbrella review can inform future research prioritization in three key ways. First, nutrients and BFCs lacking gold-standard RCT evidence will be highlighted. Second, underrepresented age groups across existing studies will be highlighted, particularly given the disproportionate focus on early childhood compared to middle childhood and adolescence [43]. Third, methodological weaknesses in existing RCTs will be identified, along with recommendations to improve the methodological rigor of future work. For example, while RCTs are the gold standard for linking specific nutrients to key outcome variables, findings may remain ambiguous or null for several reasons, including the inappropriateness of this design in particular settings, attrition, or minimal differences between the control and intervention arms [44]. This review will add nuance to and address the limitations of the RCT approach outlined by previous studies [44], enabling us to suggest alternatives for future research. Findings can serve as a starting point for researchers seeking to expand the evidence base on nutrients and BFCs related to brain health. Once evidence is established for key nutrients associated with brain health, clinical trials can be conducted to determine optimal doses and how dosage may vary across the life course. Additionally, findings can inform key national, state, and local public health priorities and subsequent interventions.

As the aging population continues to grow, maintaining brain health has become an increasingly urgent public health priority [45, 46]. To an extent, dietary intake patterns that optimize brain health represent a modifiable behavior change that can and should be advocated in public health and clinical practice. Despite advances in neuroscience, effective therapeutic options to preserve or restore brain function remain limited, underscoring the unmet need for preventive strategies.

Cumulative evidence supports a protective role of diet and nutrients in promoting brain health. In particular, higher intakes of long-chain n-3 fatty acids, folate, carotenoids, and tocopherols (vitamin E) have been associated with a reduced risk of cognitive decline among older adults [47, 48]. Moreover, adherence to dietary patterns such as the Mediterranean and DASH [3] diets has been shown not only to preserve cognitive health in later life, but also to support cardiovascular health in midlife—a key determinant of brain health in aging.

Because brain functions, metabolic demands, body composition, and pre-existing health conditions differ across developmental and aging stages, nutrient requirements for maintaining brain health are likely to vary across the lifespan. Defining these stage-specific nutritional needs is essential for effective prevention strategies. Nutrient requirements are dynamic and vary across life stages. This variability extends to nutrients and BFCs that may influence brain health. Key nutrients across life stages for brain health are provided in the Supplementary Table S2. These requirements are not static, but rather are influenced by multiple interacting factors, including but not limited to body composition [49, 50], nutrition status (over versus undernutrition) [51], gender [52], pre-existing chronic conditions [15], genetic predisposition [52], pharmacotherapy [53], and environmental exposure [54]. As a result, future efforts informed by this umbrella review should emphasize strategies that support brain health throughout the lifespan, as a cost-effective and feasible approach to managing specific nutrient requirements across the life stage.

This review will also consider the heterogeneity of the study populations, dietary assessment methods, nutrient sources (supplements vs. whole foods), and brain health outcomes, which have contributed to the inconsistent findings in literature. By evaluating both the quality and scope of existing systematic reviews, this umbrella review will identify areas of robust evidence as well as domains where further primary research is needed. In particular, the timing of nutrient exposure throughout the lifespan, whether during early life, middle age, or late life, modifies the association with brain health outcomes, which could inform precision nutrition approaches and age-specific recommendations. In addition, this umbrella review will provide a valuable resource for policymakers and researchers by mapping current evidence onto existing dietary guidelines, which will inform whether current nutrient intake recommendations are sufficient to support brain health or whether additional consideration is warranted for specific life stages. Finally, we acknowledge that caffeine is an important neuroactive dietary compound; however, it was excluded due to the methodological challenges of isolating its independent effects from complex dietary matrices, as well as the substantial heterogeneity its inclusion would introduce [30, 31]. Future research should prioritize a dedicated synthesis of caffeine and other neuroactive dietary components, given their widespread consumption and potential cognitive effects.

In conclusion, this umbrella review seeks to bridge the gap by summarizing current evidence, identifying limitations, and highlighting directions for future research. Ultimately, these efforts may support the development of evidence-based dietary strategies aimed at optimizing brain health from early life through older adulthood.

## Supplementary Information

Supplementary Material 1. PRIOR checklist.

Supplementary Material 2. AMSTAR-2 Checklist

Supplementary Material 3: Supplementary Table S1. Sample search strategy for Nourish the Mind: Protocol for an Umbrella Review on Nutrients and Bioactive Food Components Supporting Brain Health Across the Lifespan

Supplementary Material 4: Supplementary Table S2. Key Nutrients Across Life Stages for brain health*

## Data Availability

As this is a protocol, no datasets have yet been generated. The datasets used and/or analyzed in this umbrella review will be available from the corresponding author on reasonable request.

## Abbreviations

AIs: Adequate intakes
ALA: Alpha-linolenic acid
AMSTAR-2: A Measurement Tool to Assess Systematic Reviews 2
BFCs: Bioactive Food Components
CCA: Corrected covered area
COSMOS: COcoa Supplement and Multivitamin Outcomes Study
DRIs: Dietary reference intakes
EAR: Estimated average requirements
JBI: Joanna Briggs Institute
MCI: Mild Cognitive Impairment
MIND: Mediterranean-DASH Intervention for Neurodegenerative Delay
PEO: Population–Exposure–Outcome
POINTER: Protect Brain Health Through Lifestyle Intervention to Reduce Risk
PRIOR: Preferred Reporting Items for Overviews of Reviews
PROSPERO: Prospective Register of Systematic Reviews
RDAs: Recommended Dietary Allowances
SCD: Subjective Cognitive Decline
WHO: World Health Organization
