# Supplementary material for: Nourish the mind: protocol for an umbrella review on nutrients and bioactive food components supporting brain health across the lifespan
Source: Syst Rev. 2026 Jun 22;15:241. doi: 10.1186/s13643-026-03250-6 (PMC13543491; doi:10.1186/s13643-026-03250-6)
Supplement: Supplementary file 3 [file 13643_2026_3250_MOESM3_ESM.docx]

PROSPERO ID: CRD420251186632

**Supplementary Table S1.** Sample search strategy for Nourish the Mind: Protocol for an Umbrella Review on Nutrients and Bioactive Food Components Supporting Brain Health Across the Lifespan

| Database: Ovid MEDLINE(R) ALL | |
| --- | --- |
| Line | Query |
| 1 | exp cognition/ or exp cognitive science/ exp memory/ or exp attention/ or executive function/ or cognitive aging/ or mental fatigue/ or neurodevelopmental disorders/ or child development/ or exp brain/ or exp neuroimaging/ or nerve net/ or exp neurogenesis/ or Cerebrovascular Circulation/ or neuroprotection/ or exp neuronal plasticity/ or Neuroinflammatory Diseases/ or exp "Receptors, Neurotransmitter"/ or exp Neurotransmitter Transport Proteins/ or synaptic transmission/ or exp neural pathways/ or dopamine/ or serotonin/ or acetylcholine/ or gamma-Aminobutyric Acid/ or Brain-Derived Neurotrophic Factor/ |
| 2 | (Cognit* or Memory or Attention or Mental-concentration or Mental-focus or attentional-control or Executive-function* or Executive-control or Processing-speed* or Mental-processing or Psychomotor-speed* or Healthy-aging or Healthy-ageing or Mental-fatigue or mental-exhaustion or Neurodevelopment* or Neuro-development* or cerebral-development or Neurological-health or Brain or neurovascular or cerebrovascular or neuroimaging or neuro-imaging or cortical-thickness or cortex-thickness or hippocampal-volume or functional-connectivity or resting-state or neural-network* or nerve-net* or neurogenesis or cerebral-blood-flow or cerebral-circulation or cerebral-perfusion or neuroprotect* or neuro-protect* or neuron-protect* or neural-protect* or neuronal-protect* or neuroplastic* or neuro-plastic* or neural-plastic* or neuronal-plastic* or synap*-plastic* or neuroinflamm* or neuro-inflamm* or microglia or astrocyt* or astroglia or total-tau or t-tau or phosphorylated-tau or p-tau or neurofilament-light-chain or glial-fibrillary-acidic-protein or GFAP or neurotransmi* or neural-signal* or dopamine or serotonin or acetylcholine or GABA or gamma-aminobutyric-acid or BDNF or brain-derived-neurotrophic-factor).mp. |
| 3 | 1 or 2 |
| 4 | nutrients/ or dietary supplements/ or exp "fats, unsaturated"/ or exp "fatty acids, omega-3"/ or exp "fatty acids, omega-6"/ or exp "fatty acids, unsaturated"/ or exp dietary fiber/ or micronutrients/ or trace elements/ or vitamins/ or exp "vitamin b complex"/ or exp "vitamin b 12"/ or biotin/ or exp folic acid/ or inositol/ or niacin/ or pantothenic acid/ or pyridoxine/ or exp riboflavin/ or exp thiamine/ or exp "vitamin a"/ or exp ascorbic acid/ or exp "vitamin d"/ or exp "vitamin e"/ or exp "vitamin k"/ or exp choline/ or copper/ or iodine/ or iron/ or "iron, dietary"/ or magnesium/ or manganese/ or selenium/ or zinc/ or phytochemicals/ or exp carotenoids/ or terpenes/ or exp polyphenols/ or exp flavonoids/ or stilbenes/ or curcumin/ or acetylcysteine/ or exp glutathione/ or thioctic acid/ or exp probiotics/ or exp prebiotics/ or exp "fatty acids, volatile"/ or exp amino acids/ |
| 5 | (bioactive-component* or bioactive-compound* or bioactive-natural-product or (bioactive* adj10 food*) or biologically-active-food* or bioactive-ingredient* or nutrient* or dietary-supplement* or nutraceutical* or nutraceutical* or neutriceutical* or neutraceutical* or herbal-supplement* or macronutrient* or macro-nutrient* or PUFA or MUFA or polyunsaturated-fat* or monounsaturated-fat* or unsaturated-fat* or unsaturated-oil* or dietary-fat* or dietary-oil* or omega-3 or n-3-fatty-acid* or n3-fatty-acid* or ALA or EPA or DHA or linolenic-acid* or linoleic-acid* or linolenate or eicosapentaenoic-acid* or eicosapentanoic-acid* or timnodonic-acid* or docosahexaenoic-acid* or docosahexenoic-acid* or docosahexenoate or omega-6 or n-6-fatty-acid* or n6-fatty-acid* or linoleic-acid* or gamolenic-acid* or arachidonic-acid* or sodium-arachidonate or vitamin-f or long-chain-n-3 or long-chain-fatty-acid* or fiber  or fibers or fibre or fibres or resistant-starch* or micronutrient* OR micro-nutrient* or vitamin* or cobalamin* or cyanocobalamin* or biotin or folate or folic-acid or inositol  or mesoinositol or myoinositol or niacin* or nicotinamide or nicotinic-acid or pantothenic-acid or pantothenate or pyridox* or riboflavin or thiamin* or retinol or retinal or retinoi* or retinyl-ester* or beta-caroten* or carotenoid* or tretinoin or alitretinoin or ascorbic-acid or ascorbate or cholecalciferol or ergocalciferol or calcitriol or calciferol* or tocopherol* or tocotrienol* or phylloquinone or phytomenadione or phyllohydroquinone or phytonadione or menaquinone or menadione or vitamin-like or choline or copper or iodide or iodine or iron or magnesium or manganese or selenium or zinc or phytonutrient* or phyto-nutrient* or carotenoid* or tetraterpene* or astaxanthin* or beta-carotene* or betacarotene* or beta-cryptoxanthin* or lutein* or provitamin-A or terpene* or terpenoid* or isoprenoid* or zeaxanthin* or polyphenol* or provinol* or resveratrol* or tannin or tannins or flavonoid* or bioflavonoid* or anthocyani* or catechin* or epicatechin* or flavanol* or flavanone* or hesperidin or hesperetin or flavone* or flavonol* or quercetin* or stilbene* or curcumin* or turmeric or curcuminoid* or acetylcysteine* or mercapturic-acid or glutathione* or lipoic-acid or probiotic* or pro-biotic* or Lactobacillus or Bifidobacterium or prebiotic* or pre-biotic* or synbiotic* or syn-biotic* or postbiotic* or post-biotic* or short-chain-fatty-acid* or acetate or acetic-acid* or butyrate* or butyric-acid* or butanoic-acid* or propionate* or propanoate* or propionic-acid* or propanoic-acid* or amino-acid* or alanine or arginine or asparagine or aspartic-acid or cysteine or glutamic-acid or glutamine or glycine or histidine or isoleucine or leucine or lysine or methionine or phenylalanine or proline or serine or threonine or tryptophan or tyrosine or valine).mp. |
| 6 | 4 or 5 |
| 7 | "Meta-Analysis as Topic"/ or Meta-Analysis/ or exp "Systematic Reviews as Topic"/ or Systematic review/ or "Review Literature as Topic"/ or meta-analy$.tw. or metaanaly$.tw. or (systematic adj (review$1 or overview$1)).tw. or cochrane.ab. or embase.ab. or (psychlit or psyclit).ab. or (psychinfo or psycinfo).ab. or (cinahl or cinhal).ab. or science-citation-index.ab. or bids.ab. or cancerlit.ab. or reference list$.ab. or bibliograph$.ab. or hand-search$.ab. or relevant-journals.ab. or manual-search$.ab. |
| 8 | (Selection-criteria.ab. or data-extraction.ab.) and Review/ |
| 9 | 7 or 8 |
| 10 | 9 not (Comment/ or Letter/ or Editorial/) |
| 11 | 3 and 6 and 10 and (eng or english).lg. not ((exp animals/ OR exp "models, animal"/ or exp "animal experimentation"/ or veterinary.fs.) not (exp Humans/ or exp "human experimentation"/ or exp "human genetics"/)) |
| 12 | limit 11 to yr="2016 -Current" |
