# Supplementary material for: Nourish the mind: protocol for an umbrella review on nutrients and bioactive food components supporting brain health across the lifespan
Source: Syst Rev. 2026 Jun 22;15:241. doi: 10.1186/s13643-026-03250-6 (PMC13543491; doi:10.1186/s13643-026-03250-6)
Supplement: Supplementary file 4 [file 13643_2026_3250_MOESM4_ESM.docx]

**Supplementary Table S2.** Key Nutrients Across Life Stages for Brain Health*

| **Life Stage** | **Nutrient** | **Rationale** |
| --- | --- | --- |
| Toddlers  (13-23 months) | Vitamin D^1^ | Supports bone health, musculoskeletal development, and maintains calcium homeostasis^3^ |
|  | Calcium^1^ | Supports bone structure development^4^ |
|  | Iron^1^ | Supports neurologic development* and immune function ^1^ |
|  | Zinc^1^ | Supports growth and immune function^1^ |
|  | Vitamin B12^1^ | Supports brain development, neural myelination, and cognitive function*^5^ |
|  | Choline^1^ | Supports neurodevelopment, especially memory and learning*^6^ |
|  | Omega-3 and Omega-6 Fatty Acids^1^ | Support rapid brain development*^1^ |
| Children  (2-8 years) | Calcium^1^ | Supports bone structure development^4^ |
|  | Vitamin D^1^ | Supports bone health, musculoskeletal development, and maintains calcium homeostasis^3^ |
| Adolescents  (9-18 years) | Phosphorus^1^ | Binds with calcium to form strong bones and teeth and is involved in metabolism and protein synthesis, maintains homeostasis and acid-base balance^7^ |
|  | Magnesium^1^ | Supports muscle and bone metabolism, is involved in numerous enzymatic reactions, and facilitates neuromuscular function*^8^ |
|  | Choline^1^ | Optimizes bone density, maintains cell membrane integrity, and is involved in methylation reactions and lipid metabolism, precursor to neurotransmitters, suggesting involvement in cardiovascular and cognitive health*^9,10^ |
|  | Iron^1^ | Serves as a cofactor for numerous proteins and enzymatic reactions, transports oxygen, and stores oxygen in muscle for later use during contraction^11^ |
|  | Folate^1^ | Supports DNA synthesis, neural and cardiovascular development, red blood cell production, and immune function*^12^ |
|  | Vitamin B6^1^ | Supports the development of neurotransmitters and cognitive function, plays a role in mood, brain function, and energy production*^13^ |
|  | Vitamin B12^1^ | Supports enzymatic reactions and nutrient metabolism, maintains the nervous system, and synthesizes DNA*^4^ |
| Adults  (19-59 years) | Calcium^1^ | Promotes bone health, delays or prevents the onset of osteoporosis^1^ |
|  | Vitamin D^1^ | Supports calcium homeostasis and bone health, neuroimmunomodulation, regulation of neurotrophic factors, neuroprotection, and neuroplasticity*^15^ |
| Older Adults  (60+ years) | Calcium^1^ | Promotes bone health, delays or prevents the onset of osteoporosis^1^ |
|  | Vitamin D^1^ | Maintains skeletal health, involved in the prevention of chronic disease, including cognition, mental health, and cardiovascular disease*^16^ |
|  | Vitamin A^1,2^ | Supports immune function, oxidative reactions, vision, and skin integrity^17^ |
|  | Vitamin C^1,2^ | Supports brain development and neuroprotection, neutralizes free radicals, supports immune function*^18^ |
|  | Vitamin B12^1,2^ | Preserves cognitive function, prevents chronic disease, protects the immune system*^17^ |
|  | Zinc^1,2^ | Supports the immune response and cell proliferation, and is a component of enzymatic reactions^19^ |
|  | Iron^1,2^ | Supports metabolic functions, including oxygen transport, energy production, neutralizing free radicals, DNA synthesis, neurotransmitter production, and neuroprotection*^17^ |

*Note.* The Dietary Guidelines for Americans 2020-2025 life stages were used to categorize key nutrients. The life stages are as follows: Toddlers (13 - 23 months), Children (aged 2-8 years), adolescents (aged 9-18 years), adults (aged 19-59 years), women who are pregnant or lactating, and older adults (aged 60 years and above). While the Dietary Guidelines for Americans classifies infants and toddlers in the same group, from birth to age 23 months, we will exclude infants and emphasize months 13-23 with toddlers, through old age as the focus of this umbrella review is on nutrients and BFCs for brain health, and pregnancy and lactation have therefore been excluded.

* Asterisk denotes brain health function.

**References**

1. U.S. Department of Agriculture & U.S. Department of Health and Human Service. Dietary Guidelines for Americans 2020-2025. 9^th^ ed. 2020. [https://www.dietaryguidelines.gov](https://www.dietaryguidelines.gov/). Accessed 12 Feb 2026.
2. Boyle NB, Billington J, Lawton C, Quadt F, Dye L. A combination of green tea, rhodiola, magnesium and B vitamins modulates brain activity and protects against the effects of induced social stress in healthy volunteers. Nutr Neurosci. 2022 Sep 2;25(9):1845–59.
3. Stoica AB, Mărginean C. The Impact of Vitamin D Deficiency on Infants’ Health. Nutrients. 2023 Oct 16;15(20):4379.
4. Shertukde SP, Cahoon DS, Prado B, Cara KC, Chung M. Calcium Intake and Metabolism in Infants and Young Children: A Systematic Review of Balance Studies for Supporting the Development of Calcium Requirements. Adv Nutr. 2022 Sep;13(5):1529–53.
5. Venkatramanan S, Armata IE, Strupp BJ, Finkelstein JL. Vitamin B-12 and Cognition in Children. Adv Nutr. 2016 Sep;7(5):879–88.
6. Bragg MG, Prado EL, Caswell BL, Arnold CD, George M, Oakes LM, et al. The association between plasma choline, growth and neurodevelopment among Malawian children aged 6–15 months enroled in an egg intervention trial. Matern Child Nutr. 2023 Apr;19(2):e13471.
7. Park Y, Han J. Mineral Balance and Metabolic Syndrome in Adolescents: Focus on Calcium and Phosphorus Intake. Healthcare. 2021 Nov 9;9(11):1525.
8. Dong Y, Chen L, Gutin B, Huang Y, Dong Y, Zhu H. Magnesium Intake, C-Reactive Protein, and Muscle Mass in Adolescents. Nutrients. 2022 Jul 14;14(14):2882.
9. Leermakers ETM, Moreira EM, Kiefte-de Jong JC, Darweesh SKL, Visser T, Voortman T, et al. Effects of choline on health across the life course: a systematic review. Nutr Rev. 2015 Aug;73(8):500–22.
10. Li K, Guo C, Yin Y, Zhu Z. Association between dietary choline intake and bone mineral density in adolescents: a NHANES-based study. BMC Pediatr. 2025 Jul 2;25(1):512.
11. Mesías M, Seiquer I, Navarro MP. Iron Nutrition in Adolescence. Crit Rev Food Sci Nutr. 2013 Jan;53(11):1226–37.
12. M. Nair J, Chakraborty S, Kasomva K, Chauhan G, Mathur S, Basu A, et al. Unraveling folate deficiency: prevalence, biochemical associations, genetic determinants, and strategies for adolescent health in North India. Eur J Clin Nutr. 2025 Sep;79(9):847–54.
13. Herbison CE, Hickling S, Allen KL, O’Sullivan TA, Robinson M, Bremner AP, et al. Low intake of B-vitamins is associated with poor adolescent mental health and behaviour. Prev Med. 2012 Dec;55(6):634–8.
14. MacFarlane AJ, Shi Y, Greene-Finestone LS. High-Dose Compared with Low-Dose Vitamin B-12 Supplement Use Is Not Associated with Higher Vitamin B-12 Status in Children, Adolescents, and Older Adults. J Nutr. 2014 Jun;144(6):915–20.
15. Anglin RES, Samaan Z, Walter SD, McDonald SD. Vitamin D deficiency and depression in adults: systematic review and meta-analysis. Br J Psychiatry. 2013 Feb;202(2):100–7.
16. Meehan M, Penckofer S. The Role of Vitamin D in the Aging Adult. J Aging Gerontol. 2014 Jul 25;2(2):60–71.
17. Watson J, Lee M, Garcia-Casal MN. Consequences of Inadequate Intakes of Vitamin A, Vitamin B12, Vitamin D, Calcium, Iron, and Folate in Older Persons. Curr Geri Rep. 2018 Jun;7(2):103–13.
18. Harrison FE. A Critical Review of Vitamin C for the Prevention of Age-Related Cognitive Decline and Alzheimer’s Disease. JAD. 2012 Apr 16;29(4):711–26.
19. Cabrera ÁJR. Zinc, aging, and immunosenescence: an overview. Pathobiology of Aging & Age-related Diseases. 2015 Jan;5(1):25592.

1.
